# Preparation of scFv stabilized chromatosomes for single-particle cryo-EM structure determination

**DOI:** 10.1016/j.xpro.2021.100396

**Published:** 2021-03-20

**Authors:** Bing-Rui Zhou, Yawen Bai

**Affiliations:** 1 Laboratory of Biochemistry and Molecular Biology, National Cancer Institute, National Institutes of Health, Bethesda, MD 20892, USA

**Keywords:** Molecular Biology, Antibody, Protein Biochemistry, Protein expression and purification, Structural Biology, Cryo-EM

## Abstract

The chromatosome, a nucleosome bound to a histone H1, is the structural unit of metazoan chromatin. Determination of the high-resolution structure of the chromatosome is challenging due to the dynamic nature of H1 binding. Here, we present a protocol for purifying an optimized single-chain antibody variable fragment (scFv) that can be used to stabilize the chromatosome for single-particle cryo-EM studies. This protocol facilitates high-resolution cryo-EM structure determination of nucleosomes with a natural DNA sequence, chromatosomes, and other protein nucleosome complexes.

For complete details on the use and execution of this protocol, please refer to Zhou et al. (2021).

## Highlights

• Optimization of nucleosome antibody fragment (scFv) construct to increase its stability
• Strategy for refolding and purification of the scFv from the inclusion body
• Use of scFv to facilitate single-particle cryo-EM of the protein nucleosome complex

## Graphical Abstract

**Figure:**
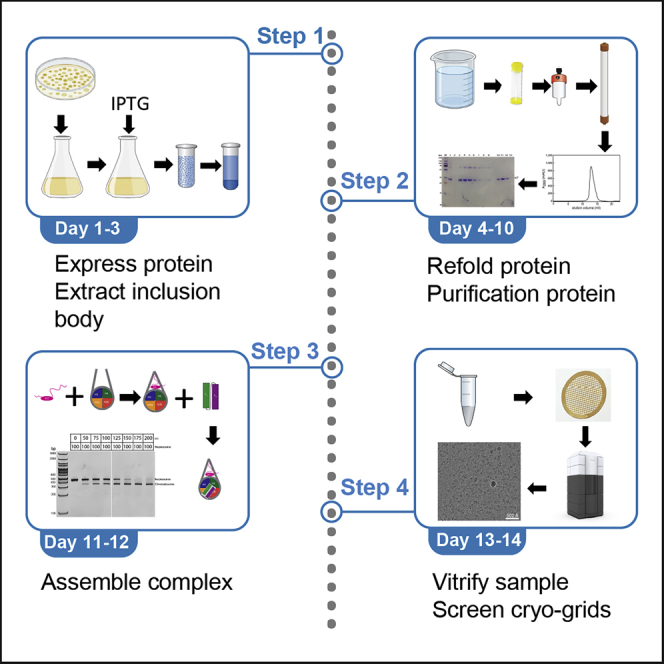

## Before you begin

**Figure 1:**
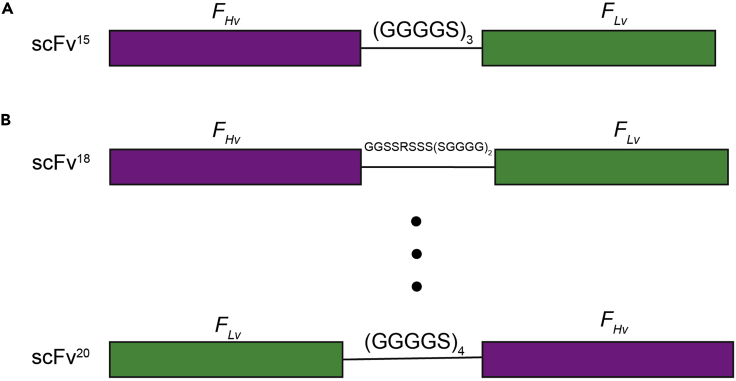
Schematic illustration of the design of the single-chain variable fragment (scFv) region of the *PL 2-6* nucleosome antibody Purple box: fragment of Heavy chain (*F*_*Hv*_), green box: fragment of Light chain (*F*_*Lv*_), connection line: flexible linker between the two chains. (A) original scFv construct. (B) redesign of scFv constructs.

We previously demonstrated that a single-chain variable fragment (scFv) of a nucleosome antibody (Zhou et al., 2019) can stabilize the nucleosome with a natural DNA sequence, which enabled us to determine the cryo-EM structure of a native-like nucleosome to a global resolution of 2.6 Å. However, the original scFv construct (scFv^15^) (Figure 1A) is not stable and gradually forms aggregations when stored at 4°C (note, scFv^15^ cannot be stored in frozen form as it completely aggregates upon thawing). Here we optimized the construct by switching the order of Hv and Lv fragments or by changing the length of the flexible linker (Krebber et al., 1997) (Figure 1B). Remarkably, we found that one of the constructs (scFv^20^) is much more stable. In our hands, scFv^20^ can be stored at 4^o^C for nearly a year without forming any apparent aggregations. In this protocol, scFv represents this optimized scFv^20^, unless stated otherwise.

### Reconstitution of nucleosome with 197 bp DNA

**Timing: 5–7 days**

For this protocol, one should prepare four core histones following the previously published protocol (Dyer et al., 2004). Below are the steps for purifying the 197 bp W601 DNA from a plasmid (pUC19-16x197_W601) and using it for nucleosome reconstitution.

### Preparation of 197bp W601 DNA

**CRITICAL:** A plasmid with an array of repeat sequences tends to be unstable when propagated in bacterial strains, therefore it is critical to utilize a stable cell line such as NEB Stable cells or Invitrogen One Shot stbl2 cells. After transformation, check the plasmid isolated from several colonies by restriction enzyme digestion or DNA sequencing. This analysis is equally critical for the production of the desired DNA with high yield.

1. Transform 1 **μ**L of pUC19-197x16_W601 plasmid (∼100 ng/μL) into 50 μL NEB Stable cells following the manufacturer’s protocol and spread around ∼20 μL cells onto an LB agar plate containing 100 ug/mL ampicillin. Incubate the plate in a 37°C incubator for ∼18 h.
2. Label 4 colonies separately, then inoculate each colony into 10 mL of LB medium containing 100 ug/mL ampicillin in a 50 mL Falcon tube. Grow in a 37°C incubator shaker at 220 rpm for 6 - 8 h.
3. Transfer 4 mL of cell culture from each tube to four new Falcon tubes. Extract plasmids using the Promega Mini-Prep Kit, following the manufacturer’s protocol.
4. Digest 200 ng of each purified plasmid using PstI and EcoRI restriction enzymes, which should yield a 3.2 kb insert (16 × 197 bp) band and a 2.7 kb pUC vector band when resolved in a 2% agarose gel (Figure 2, left, lanes 1–4). Plasmid digested with SmaI restriction enzyme alone should produce a 197 bp W601 DNA band and 2.9 kb vector band (Figure 2, right, lanes 1–4).

**Figure 2:**
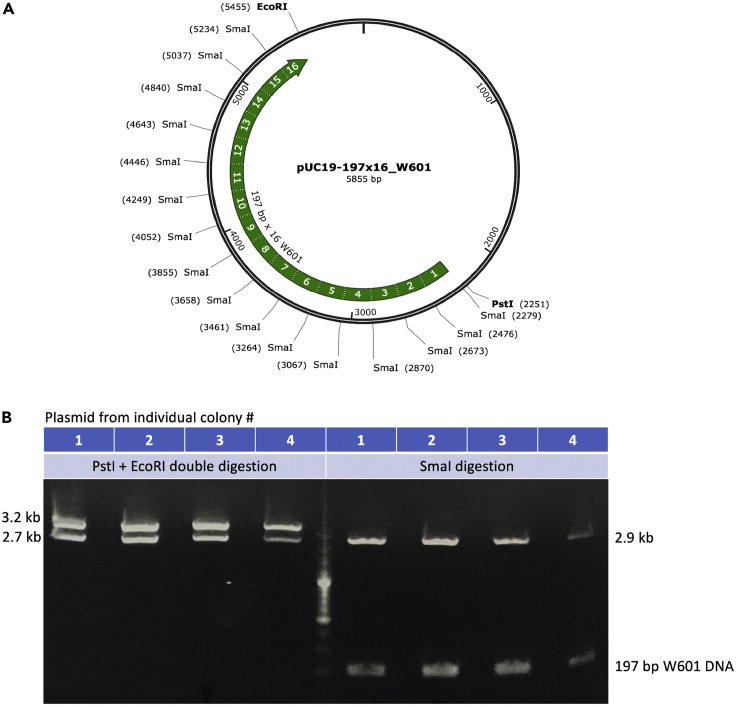
Verification the plasmids after transformation (A) A diagram of the plasmid map and restriction enzyme sites. Map is created by SnapGene. (B) Purified plasmids digested with restriction enzymes and run on a 2% agarose gel.
5. Once the plasmids are verified, the corresponding cell cultures can be combined and equally distributed to six flasks containing 1 L 2x YT cell culture medium. Incubate the cell cultures in a 37°C incubator shaker set at 250 rpm for ∼18–24 h.
6. Harvest the cells by centrifugation at 4000x*g*, 4°C for 30 min in an Eppendorf 5810R centrifuge. Perform large scale plasmid preparation by following the published protocol (Dyer et al., 2004).
7. The typical yield of plasmid from 6 L of cell culture is ∼80 - 120 mg. To release the 16 copies of 197 bp W601 DNA, digest the plasmid with 10 U SmaI restriction enzyme per mg plasmid in 1x NEB Cutsmart buffer, adjust the plasmid concentration to ∼2 mg/mL with deionized (DI) water, and incubate in a 37°C incubator for 18 h.
8. Analyze the SmaI plasmid digestion on a 2% agarose gel. The presence of multiple bands other than the 197 bp DNA and 2.9 kb plasmid DNA suggests that the digestion is incomplete. In this case, add 50% more enzyme and incubate another 18 h.
9. Once the digestion is complete, Aliquot to ∼20 mL in several 50 mL Falcon tubes. Precipitate the plasmid backbone (2.9 kb band) by stepwise adding 40% PEG 6000 to the digestion solution ranging from 4% to 6%, while simultaneously adding 4 M NaCl to a final concentration of 0.5 M. Mix well. When the mixture turns cloudy, place the tubes on ice for at least 1 h to further precipitate the plasmid backbone DNA.
10. Pellet the plasmid backbone DNA by centrifugation at 4000x*g*, 4°C for 30 min in an Eppendorf 5810R centrifuge. Decant ∼15 mL of supernatant to each 50 mL Falcon tube and precipitate the 197 bp DNA by adding ∼30 mL of cold ethanol.
11. Pellet the 197 bp DNA by centrifugation at 4000x*g*, 4°C for 30 min in an Eppendorf 5810R centrifuge, discard the supernatant, wash the pellet with 70% ethanol three times, air dry the pellet and then redissolve it in TE 10/1 (10 mM Tris, 1 mM EDTA, pH8.0) buffer.
12. Extract the DNA once with phenol:chloroform:isoamyl alchohol (25:24:1) (PCIA) and then with chloroform:isoamyl alcohol (CIA) to remove residual enzyme and PEG 6000. Precipitate the DNA using ethanol, and then redissolve it in TE 10/1 buffer. The typical yield of 197 bp DNA is ∼30 - 40 mg. Store the 197 bp DNA at 4^o^C at a concentration of ∼ 5 mg/mL.

***Alternatives:*** DNA can also be prepared using a large-scale PCR method as detailed in a recent protocol (Nodelman et al., 2020).

### Reconstitution of nucleosomes with 197 bp DNA

13. Dissolve four human core histones (H2A, H2B, H3, and H4) in freshly made histone denaturation buffer (100 mM Tris-HCl, pH 8.0, 7 M guanidine, 1 mM EDTA and 5 mM DTT) by gently rocking at 25°C for ∼ 1 h.
14. Mix H2A, H2B, H3, and H4 solutions at a molar ratio of 1.2:1.2:1:1, and then dialyze against 5 L histone octamer refolding buffer (10 mM Tris-HCl, pH 8.0, 2 M NaCl, 1 mM EDTA, and 5 mM 2-mercapitolethanol) using 6 - 8 kDa cut-off dialysis tubing at 4°C for 18 h.
15. Concentrate the refolding solution to ∼2 mL using an Amicon Ultra 30K MWCO centrifugal filter unit spin at 4000x*g*. Purify the histone octamer using a Superdex 200 10/60 gel filtration column that has been pre-equilibrated with refolding buffer.
16. Mix the purified histone octamer with 197 bp DNA at a molar ratio of 1:1.2 in refolding buffer. Reconstitution of the nucleosome essentially follows a previous protocol (Dyer et al., 2004).

**CRITICAL:** It is difficult to separate the H3/H4 tetramer peak from the histone octamer peak using a Superdex S200 column. Using a molar ratio of 1.2:1.2:1:1 for H2A, H2B, H3, and H4, instead of 1:1:1:1, increases the folding efficiency of the histone octamer and limits the formation of H3/H4 tetramers.

### Preparation of recombinant H1 and its chaperone ProTα

**Timing: 3–4 days**

H1 and ProTα genes were codon-optimized, commercially synthesized and cloned into the pET42b or pET28b vector using NdeI and BamHI restriction sites. In order to purify the full-length recombinant protein using Ni-NTA beads, we added an in-frame his6 tag at the C-terminus of H1 and ProTα.

### Expression of H1 and ProTα in bacterial cell

17. Transform 1 μL of pET42b-H1 or pET28-ProTα plasmid into 20 μL BL21(DE3) RIPL cells following the manufacturer’s protocol. Spread ∼10 μL of transformed cells onto an LB agar plate containing 50 ug/mL kanamycin. Incubate the plate in a 37°C incubator for ∼18 h.
18. Inoculate a single colony into 10 mL LB medium containing 50 μg/mL kanamycin and grow in a 37°C incubator shaker at 220 rpm for 16 h.
19. Dilute the 10 mL cell culture into 1 L LB medium containing 50 μg/mL kanamycin. Continue to grow until the OD_600_ reaches 0.6–0.8. Add 0.5 mM IPTG to induce protein expression for 3 h.
20. Harvest the cells by centrifugation at 3000x*g*, 10 min at 4°C in a Beckman Coulter Avanti J-20I centrifuge. Store the cell pellet at −80°C until the next step.

### Purification of recombinant H1 and ProTα

21. Resuspend the cell pellet in 50 mL lysis buffer (50 mM sodium phosphate, 8 M urea or 4 M guanidine, 0.75 M NaCl and 5 mM imidazole, pH 8.0). Lyse the cells using a Misonix sonicator (15 s ON, 1 min OFF, 15% power level, total 4 min).
22. Clear the cell lysate by centrifugation in a Beckman Coulter ultracentrifuge at 130,000x*g*, 20°C for 1 h.
23. Mix the cleared lysate with 2 mL of Ni-NTA beads in an Econo column. Gently rock the column for 1 h.
24. Let the lysate flow through the column. Perform stepwise washes of the Ni-NTA beads using 10 mL of wash buffer (50 mM sodium phosphate, 6 M urea, 0.5 M NaCl, pH 8.0) containing 5, 10, 15, 20, 25 mM imidazole.
25. Elute the bound protein using elute buffer (50 mM sodium phosphate, pH 8.0, 6 M urea, 0.5 M NaCl, 250 mM imidazole) five times, 8 mL each. Check each elution fraction and the flow through on an SDS PAGE gel.
26. Combine the fractions that contain protein, inject into a protein RP HPLC column, and elute the protein by linearly increasing the acetonitrile concentration from 10% to 60% using a Waters HPLC system.
27. Freeze the elution peak containing pure protein on dry ice and lyophilize the protein using a freeze dryer.

***Note:*** Lyophilized protein powder is stable for years and can be stored either at 22°C (avoid direct light) or at −80°C.

***Alternatives:*** If an HPLC instrument is not available, proteins from step 25 can be buffer exchanged into low salt buffer (50 mM sodium phosphate, 6 M urea, 0.15 M NaCl, pH 8) by dialysis and injected into a Hitrap SP column (for H1) or Hitrap Q column (for ProTα). Elute protein by linearly increasing the salt concentration from 0.15 to 0.6 M using an AKTA FPLC. Finally, remove all the buffer and salt components by thoroughly dialyzing against DI water, then lyophilize and store H1 and ProTα as described above.

## Key resources table

**Table:** 

REAGENT or RESOURCE	SOURCE	IDENTIFIER
**Bacterial and virus strains**		

BL21(DE3) RIPL codon plus	Agilent	Cat#230280
NEB Stable cells	NEB	Cat#C3040I

**Critical commercial assays**		

HiTrap SP HP column	Cytiva	Cat#17115101
HiTrap Q HP column	Cytiva	Cat#17115301
HisTrap HP His tag protein purification column	Cytiva	Cat#17524801
Protein-RP column	YMC America	Cat#PR99S05-2520W
TSKgel DEAE-5PW column	TOSOH	Cat#0007574
Superose 6 10/300 GL column	Cytiva	Cat#29091596
Superdex 200 16/600 column	Cytiva	Cat#17106901
Superdex 75 10/300 increase column	Cytiva	Cat#29148721
Ni-NTA agarose	QIAGEN	Cat#30230
SP Sepharose Fast Flow resin	Cytiva	Cat#17072901
0.22 μm Millex-GP PES membrane	Millipore	Cat#SLGP033RS
Amicon Ultra 10K MWCO centrifugal filter unit	Millipore	Cat#UFC901024
Amicon Ultra 30K MWCO centrifugal filter unit	Millipore	Cat#UFC903024
Quantifoil 1.2/1.3 holy carbon copper grids	EMS	Cat#Q250-CR1.3

**Chemicals, peptides, and recombinant proteins**		

L-Arginine	Millipore Sigma	Cat#A5006
L-Glutathione oxidized	Millipore Sigma	Cat#G4376
Phenol:chloroform:isoamyl alcohol 25:24:1	Millipore Sigma	Cat#P2069
Chloroform:isoamyl alcohol 24:1	Millipore Sigma	Cat#C0549
Imidazole	Millipore Sigma	Cat#5513
1,4-Dithioerythritol (DTE)	Millipore Sigma	Cat#D8255
LB medium	IPM Scientific	Cat#11006-004
2x YT medium	IPM Scientific	Cat#11006-055
S. O. C. medium	Invitrogen	Cat#46-0821
1 M Tris, pH 7.4	KD Medical	Cat#RGF-3340
1 M Tris, pH 8.0	Quality Biological	Cat#351-027-101
10x PBS	KD Medical	Cat#RGF-3210
Triton X-100	Millipore Sigma	Cat#X100
EDTA, 0.5 M	Quality Biological	Cat#351-027-101
Sodium chloride	Millipore Sigma	Cat#S3014
Urea	Millipore Sigma	Cat#U5378
Guanidine hydrochloride	Invitrogen	Cat#15502016
L-Arginine	Millipore Sigma	Cat#A4474
L-Glutathione oxidized	Millipore Sigma	Cat#G4376
DTT	GoldBio	Cat#DTT10
0.5 M Tris(2-carboxyethyl)phosphine hydrochloride (TCEP)	Millipore Sigma	Cat#646547
10X TBE buffer	Fisher Scientific	Cat#AC327372500
40% Acrylamide/bis solution, 29:1	Bio-Rad	Cat#1610146
Ammonium persulfate (APS)	Bio-Rad	Cat#1610700
TEMED	Bio-Rad	Cat#1610800
Isopropyl-b-D-thiogalactopyranoside (IPTG)	Fisher Scientific	Cat#BP1620
Kanamycin	Fisher Scientific	Cat#BP906
Ampicillin	Millipore Sigma	Cat#A0166
Lysozyme	Millipore Sigma	Cat#L6876

**Recombinant DNA**		

pET42b-H1.4-his6	Zhou et al., 2021	N/A
pET28b-YProTa-his6	Feng et al., 2018	N/A
pET22b-H2A	Zhou et al., 2021	N/A
pET42b-H2B	Zhou et al., 2021	N/A
pET21b-H3	Zhou et al., 2021	N/A
pET21b-H4	Zhou et al., 2021	N/A
pUC19-16x197_W601	Zhou et al., 2021	N/A
pETHis6TEV-scFv	This paper	N/A

**Software and algorithms**		

SerialEM v 3.7	Mastronarde, 2005	https://bio3d.colorado.edu/SerialEM/
SnapGene v 5.2.4	GSL Biotech LLC	https://www.snapgene.com/

**Other**		

Econo-Column chromatography column	Bio-Rad	Cat#7375021
Promega Miniprep Kit	Promega	Cat#A1460
Empty gel cassettes, mini, 1.0 mm	Thermo Fisher Scientific	Cat#NC2010
B-per Bacterial Protein Extraction Reagent	Pierce, Thermo Scientific	Cat#78243
Midori Green Advance stain solution, 20,000x	Bulldog Bio	Cat#MG04
BenchWaver 3D Rocker	Benchmark Scientific	Cat#B3D5000

***Note:*** The pET His6 TEV cloning vector with BioBrick polycistronic restriction sites (9B) was a gift from Scott Gradia (Addgene plasmid # 48284 ; http://n2t.net/addgene:48284 ; RRID:Addgene_48284), In this protocol, we didn’t remove the 6x His tag from the scFv.

## Materials and equipment

### Lysis buffer

Cell lysis buffer contains 50 mM Tris-HCl, pH7.5, 100 mM NaCl, 1 mM EDTA. Add the reagents below to 800 mL DI water:

**Table:** 

Reagent	Amount	Final concentration
1 M Tris-HCl, pH 7.4	50 mL	50 mM
0.5 M EDTA	2 mL	1 mM
NaCl	5.8 g	100 mM

Adjust pH to 7.5 at 25°C, add DI water up to 1 L. The buffer can be stored at 4°C for one month.

### Wash buffer

Inclusion body wash buffer contains 50 mM Tris-HCl, pH7.5, 100 mM NaCl, 1 mM EDTA, 1% Triton X-100. Add the reagents below to 800 mL DI water:

**Table:** 

Reagent	Amount	Final concentration
1 M Tris-HCl, pH 7.4	50 mL	50 mM
0.5 M EDTA	2 mL	1 mM
NaCl	5.8 g	100 mM
Triton X-100	10 mL	1%

Adjust pH to 7.5 at 25°C, add DI water up to 1 L. The buffer can be stored at 4°C for one month.

### Denaturation buffer

Inclusion body denaturation buffer contains 100 mM Tris–HCl, pH 8.0, 6 M Guanidine, 2 mM EDTA and 10 mg/mL 1,4-Dithioerythritol. Add the reagents below to 80 mL DI water:

**Table:** 

Reagent	Amount	Final concentration
1 M Tris-HCl, pH 8.0	10 mL	100 mM
0.5 M EDTA	0.4 mL	2 mM
Guanidine	57.3 g	6 M

Adjust pH to 8.0 at 25°C, add DI water up to 100 mL. The buffer can be stored at 4°C for one month.

***Note:*** 1,4-dithioerythritol (DTE) should be added to the buffer just before use.

### Refolding buffer

Refolding buffer contains 100 mM Tris–HCl, pH 9.5, 0.5 M arginine, 1 mM EDTA and 551 mg/L oxidized glutathione. Add the reagents below to 800 mL DI water:

**Table:** 

Reagent	Amount	Final concentration
1 M Tris-HCl, pH 8.0	100 mL	100 mM
0.5 M EDTA	2 mL	1 mM
L-arginine	87.1 g	0.5 M

Adjust pH to 9.5 at 25°C, add DI water up to 1 L. The buffer should be chilled to 10°C one day before use.

***Note:*** Oxidized glutathione powder should be added to the buffer just before use.

### Dialysis buffer

Dialysis buffer contains 20 mM Tris–HCl, pH 7.4, 100 mM urea. Add the reagents below to 4 L DI water:

**Table:** 

Reagent	Amount	Final concentration
1 M Tris-HCl, pH 7.4	90 mL	20 mM

Adjust pH to 7.4 at 25°C, add DI water up to 4.5 L. The buffer should be chilled to 4°C one day before use.

***Note:*** 30 g urea should be added to the buffer just before use.

### SP binding buffer

SP binding buffer contains 20 mM Tris–HCl, pH 7.4. Add the reagents below to 800 mL DI water:

**Table:** 

Reagent	Amount	Final Concentration
1 M Tris-HCl, pH 7.4	20 mL	20 mM

Adjust pH to 7.4 at 25°C, add DI water up to 1 L. The buffer can be stored at 4°C for one month.

### SP elution buffer

SP elution buffer contains 20 mM Tris–HCl, pH 7.4, 300 mM NaCl. Add the reagents below to 800 mL DI water:

**Table:** 

Reagent	Amount	Final Concentration
1 M Tris-HCl, pH 7.4	20 mL	20 mM
NaCl	17.5 g	300 mM

Adjust pH to 7.4 at 25°C, add DI water up to 1 L. The buffer can be stored at 4°C for one month.

### Histrap binding buffer

Histrap binding buffer contains 20 mM Tris–HCl, pH 7.4, 40 mM Imidazole, 300 mM NaCl. Add the reagents below to 80 mL DI water:

**Table:** 

Reagent	Amount	Final concentration
1 M Tris-HCl, pH 7.4	20 mL	20 mM
NaCl	17.5 g	300 mM
Imidazole	2.7 g	40 mM

Adjust pH to 7.4 at 25°C, add DI water up to 1 L and filter the buffer using a 0.22 μm filter unit. The buffer can be stored at 4°C for one month.

### Histrap elution buffer

Histrap elution buffer contains 20 mM Tris–HCl, pH 7.4, 500 mM Imidazole, 300 mM NaCl. Add the reagents below to 80 mL DI water:

**Table:** 

Reagent	Amount	Final concentration
1 M Tris-HCl, pH 7.4	20 mL	20 mM
NaCl	17.5 g	300 mM
Imidazole	34 g	500 mM

Adjust pH to 7.4 at 25°C, add DI water up to 1 L and filter the buffer using a 0.22 μm filter unit. The buffer can be stored at 4°C for one month.

### Gel-filtration buffer A

Gel-filtration buffer A contains 20 mM Tris-HCl, pH 7.4, 1 mM EDTA and 150 mM NaCl. Add the reagents below to 800 mL DI water:

**Table:** 

Reagent	Amount	Final concentration
1 M Tris-HCl, pH 7.4	20 mL	20 mM
0.5 M EDTA	2 mL	1 mM
NaCl	8.8 g	150 mM

Adjust pH to 7.4 at 25°C, add DI water up to 1 L and filter the buffer using a 0.22 μm filter unit. The buffer can be stored at 4°C for one month.

### Gel-filtration buffer B

Gel-filtration buffer B contains 20 mM Tris-HCl, pH 7.4, 1 mM EDTA and 600 mM NaCl. Add the reagents below to 800 mL DI water:

**Table:** 

Reagent	Amount	Final concentration
1 M Tris-HCl, pH 7.4	20 mL	20 mM
0.5 M EDTA	2 mL	1 mM
NaCl	35 g	600 mM

Adjust pH to 7.4 at 25°C, add DI water up to 1 L and filter the buffer using a 0.22 μm filter unit. The buffer can be stored at 4°C for one month.

### 10% APS stock

**Table:** 

Reagent	Amount	Final concentration
APS	1 g	10 % (v/w)
ddH_2_O	Up to 10 mL	n/a

Aliquot to ∼0.2 mL per Eppendorf tube, store at −20°C in frozen form.

### 4.8% native PAGE gel

To make one 4.8% native PAGE gel, combine the reagents below in a 50 mL Falcon tube:

**Table:** 

Reagent	Amount	Final concentration
ddH_2_O	8.5 mL	n/a
10x TBE	0.2 mL	0.2x
40% Acrylamide/Bis Solution, 29:1	1.2 mL	4.8%
10% APS	100 μL	0.1%
TEMED	10 μL	0.1%
**Total**	**10 mL**	**n/a**

Before adding TEMED, mix the gel solution well by gently inverting the tube a few times, then add TEMED and mix again. Pour the gel solution into an empty gel cassette on a gel casting stand, knock the cassette gently if there are air bubbles in the gel, and insert the comb tightly. Let the gel solidify for 1 h at 25°C. Gels can be stored at 4°C within a sealed plastic bag for a month. Keep the gel wet by adding a few mL of DI water or 0.2× TBE buffer to the bag.

**CRITICAL:** Acrylamide/Bis Solution is toxic if it comes in contact with skin and is harmful if inhaled. Handle all solutions with gloves. TEMED is toxic and volatile, add it to the gel solution in a chemical fume hood.

### TEN10 buffer

TEN10 Buffer contains 10 mM Tris-HCl, pH 7.4, 1 mM EDTA, 10 mM NaCl and 0.5 mM TCEP. Add the reagents below to 90 mL DI water:

**Table:** 

Reagent	Amount	Final Concentration
1 M Tris-HCl, pH 7.4	1 mL	10 mM
0.5 M EDTA	0.2 mL	1 mM
5 M NaCl	0.2 mL	10 mM
0.5 M TCEP	0.1 mL	0.5 mM

Adjust pH to 7.4 at 25°C, add DI water up to 100 mL and filter the buffer using a 0.22 μm filter unit. The buffer can be stored at 4°C for 14 days.

## Step-by-step method details

### Refolding and purification of scFv from inclusion body

**Timing: 9–10 days**

**Figure 3:**
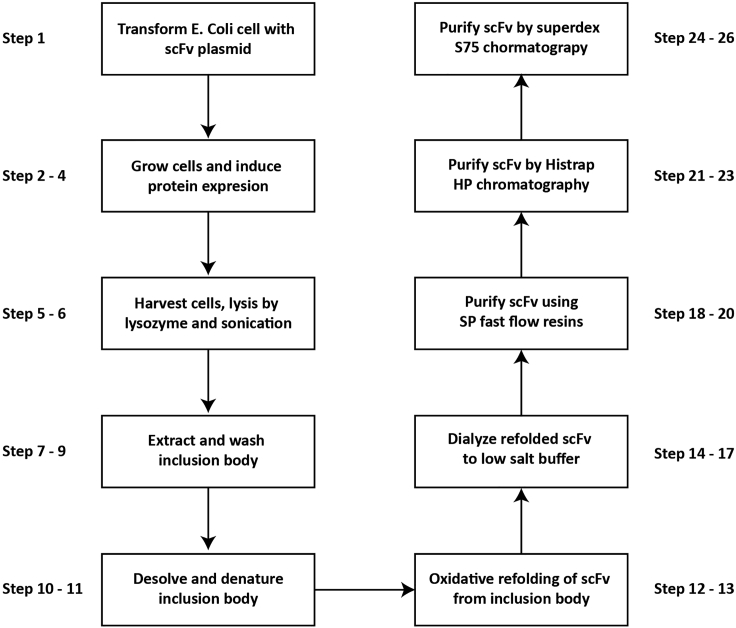
Diagram of the workflow for refolding and purification of scFv from the inclusion body

Folded scFv is stabilized by four disulfide bonds. Recombinant scFv often forms inclusion bodies in *E. coli* cells due to the reducing environment in the bacterial cytoplasm. We have tested scFv expression in NEB SHuffle T7 Competent *E. coli* cells that provide an oxidative environment, however scFv expression still results in inclusion body formation. Here we describe the refolding and purification of scFv from the inclusion body (Figure 3), with modifications of the previous protocol (Pastan and Ho, 2010) .

### Preparation of scFv from the inclusion body

1. Transform 1 μl pETHis6TEV-scFv plasmid into 20 μl BL21(DE3) RIPL cells following the manufacturer’s protocol. Spread ∼20 μL transformed cells onto an LB agar plate containing 100 μg/mL ampicillin. Incubate the plate at 37°C for 18 h.
2. Inoculate a single colony into 10 mL LB medium containing 100 μg/mL ampicillin and grow in a 37°C incubator shaker at 220 rpm for 14 h.
3. Dilute the 10 mL LB cell culture into 1 L of 2x YT medium containing 100 μg/mL ampicillin. Continue to grow until the OD_600_ of the cell culture reaches 0.8.
4. Cool the cell culture and the incubator shaker to 25°C. Induce protein expression by adding 0.5 mM IPTG and continue to grow the cells in the 25°C incubator shaker at 220 rpm for 16 h.
5. Harvest the cells by centrifugation at 3000x*g*, 10 min at 4°C in a Beckman Coulter Avanti J-20I centrifuge. Resuspend the cells in 35 mL Lysis buffer (50 mM Tris-HCl, pH7.5, 100 mM NaCl, 1 mM EDTA). Add 0.5 mL 50 mg/mL lysozyme stock solution and freeze the cells on dry ice.
6. Thaw the cells in a 42°C water bath. The cell lysate should be very sticky. Break up the sticky cell lysate using a Misonix sonicator for 4 min (15 s ON, 1 min OFF with 15% power level), keeping the sample on ice.
7. Pellet the inclusion body by centrifugation at 27,000x*g* for 20 min at 4°C in a Beckman Coulter Avanti J-20I centrifuge.
8. Discard the supernatant, resuspend the pellet in wash buffer (50 mM Tris-HCl, pH7.5, 100 mM NaCl, 1 mM EDTA, 1% Triton X-100). If the pellet is still sticky, break it up with sonication for 30 s. Collect the inclusion body pellets by centrifugation. Decant the supernatant.
9. Wash the pellet two more time using lysis buffer and collect the inclusion body pellets as above.

**Pause point:** The scFv inclusion body pellet can be stored at −80°C up to a month at this step.

***Optional:*** Inclusion bodies can be prepared from the bacterial cells using a commercially available reagent such as B-per Bacterial Protein Extraction Reagent (Pierce; Thermo Scientific Life Science, Rockford, IL, USA). Add 2.5 mL (official document states 4 mL) of the reagent containing 500 ug of lysozyme (10 μL of 50 mg/mL stock) and 20 U of DNase I (8 μL of 2500 U/mL (∼1 mg/mL) stock) per 1 g of wet cells to the bacterial cell pellet. After gentle shaking for 10 min, collect the pellets by centrifugation at 14,500x*g* for 20 min, and then suspend in 15 mL of B-per Reagent diluted 10 fold. After gentle shaking for 10 min, collect the inclusion body pellets by centrifugation at 14,500x*g* for 20 min.

### Refolding of scFv from inclusion body

10. Dissolve the inclusion body pellet with 10 mL denaturation buffer (100 mM Tris-HCI, pH8, 6 M Guanidine buffer, 2 mM EDTA). Add 1,4-dithioerythritol (DTE) powder to a final concentration of 10 mg/mL. Break up the pellet briefly using a ceramic rod and shake with a BenchWaver 3D Rocker at 66 rpm for 16 h.
11. Clear the solution by centrifugation at 130,000x*g*, 10°C for 1 h in a Beckman Coulter ultracentrifuge.
12. Add oxidized glutathione powder fresh to the 10°C chilled scFv refolding buffer (100 mM Tris–HCl, 1 mM EDTA, 0.5 M arginine, pH 9.5) to a final concentration of 551 mg/L. Stir the buffer with a magnetic stirring bar at 600 rpm. Quickly add 10 mL of the supernatant from step 11 to 1 L of refolding buffer in a glass beaker and continue stirring the buffer for 2–3 min.
13. Cover the beaker with aluminum foil. Incubate the refolding solution in a 10°C refrigerator for ∼48 h without stirring.
14. Prepare 2 x 4.5 L dialysis buffer (20 mM Tris-HCl, pH7.4) and chill to 4°C.
15. Add 30.6 g urea powder to the dialysis buffer, dialyze 1 L of refolding solution against 4.5 L dialysis buffer using 6 - 8 kDa cut-off dialysis tubing for 16 h.
16. Replace with fresh dialysis buffer, add 30.6 g urea powder and again perform dialysis for 16 h.

### Purification of scFv

**Figure 4:**
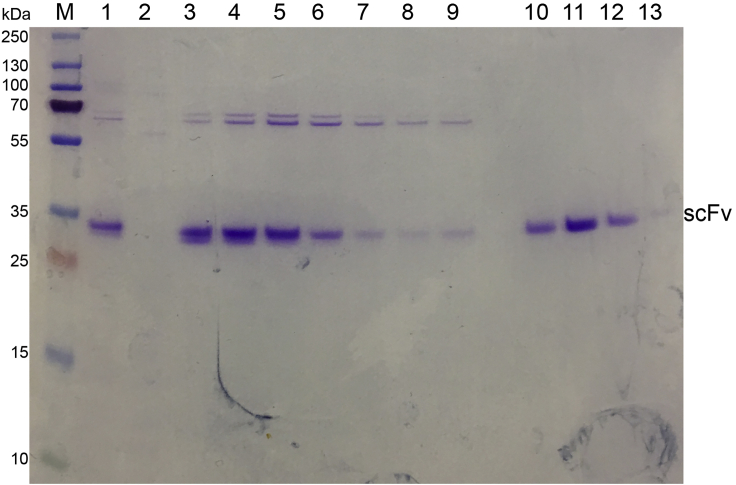
SDS PAGE analysis of scFv purification using Histrap HP and Superdex S75 column M: Thermo Scientific PageRuler Prestained Protein Ladder, 10 to 180 kDa; lane 1: samples injected into Histrap column; lane 2: flow through of Histrap column; lane 3–9: elution peak fractions from Histrap column; lane 10–13: peak fraction from Superdex S75 size exclusion column.

17. Collect all of the refolding solution in a 2 L beaker, adjust the pH to around 7.8 (add ∼2.6 mL 35% HCl) at 4°C and remove any aggregates by centrifugation at 4000x*g*, 4°C for 10 min. Filter the supernatant through a 0.22 μm filter unit.
18. Equilibrate 4 mL SP Sepharose Fast Flow resin with 20 mL of SP binding buffer (20 mM Tris–HCl, pH 7.4), 20 mL volumes of SP binding buffer with 1 M NaCl and then 20 mL volumes of SP binding buffer again. Add the resin directly into the scFv refolding solution, stirring the solution using a magnetic stir bar at 150 rpm. Let scFv bind to the SP resin for 1 h at 4°C.
19. Pass the solution through an Econo column, save the flow through temporarily. Wash the resin in the column with a total of 150 mL SP binding buffer (10 mL, 15 times), then wash the resin twice using binding buffer containing 100 mM NaCl. Elute the scFv with 48 mL SP elute buffer (20 mM Tris–HCl, pH 7.4, 300 mM NaCl, 8 mL each, 6 times). Check the OD_280_ of each elution fraction (the OD_280_ for the second elution fraction should be around 0.3 - 0.6). Troubleshooting 1
20. Analyze each fraction and the flow through on an SDS PAGE gel. The flow through can be discarded if there is no more scFv in it. The protein yield after SP chromatography is ∼10 - 15 mg.
21. Equilibrate a 5-mL Histrap column with 5 column volumes of Histrap wash buffer (20 mM Tris–HCl, pH 7.4, 40 mM Imidazole, 300 mM NaCl), 5 column volumes of Histrap elution buffer (20 mM Tris–HCl, pH 7.4, 500 mM Imidazole, 300 mM NaCl) and then 5 column volumes of Histrap wash buffer again.
22. Combine all fractions from step 19 that contain scFv, add 1/10 v/v of Histrap elution buffer to the sample (final imidazole concentration should be around 44 mM). Inject the sample into a 5 mL pre-equilibrated Histrap column, wash with 5 volumes of Histrap wash buffer. Elute by linearly increasing the imidazole concentration from 40 mM to 250 mM over 20 column volumes using an AKTA FPLC.
23. Collect fractions from the main elution peak (appears around 80 mL). Check each fraction on an SDS PAGE gel (Figure 4, lanes 3–9). The protein yield after Histrap chromatography is ∼5 - 10 mg Combine the fractions containing scFv and concentrate them to around 4 mL.

**CRITICAL:** Do not over concentrate the scFv as it will aggregate at concentration above 5 mg/mL.

24. Equilibrate the Superdex S75 10/600 column with Gel-Filtration buffer A (20 mM Tris-HCl, pH7.4, 150 mM NaCl, 1 mM EDTA) for 2 column volumes.
25. Inject 2 mL of the scFv sample from step 23 to the Superdex S75 10/600 column with a flow rate of 1 mL/min. The scFv with correct disulfide bonds appears at around 65 mL. Check each fraction on an SDS PAGE gel (Figure 4, lanes 10–13). Troubleshooting 2
26. Concentrate the peak fractions to ∼1.5 mL volume using a 10 kDa cut-off Amicon concentration device. Keep the concentration to around 5 uM, as a higher concentration can lead to aggregation and loss of scFv. The protein yield after S75 chromatography is ∼3 - 5 mg. Store scFv in a 4°C refrigerator. Troubleshooting 3

### Preparation of the scFv stabilized chromatosome for cryo-EM study

**Timing: [2 - 3 days]**

To prepare the scFv stabilized chromatosome, we first do small volume titration of 197 bp nucleosome with linker histone in the presence of chaperone ProTα and analyze each titration on a native PAGE gel. After the best ratio is determined, we then do large scale preparation of chromatosomes, and add scFv to the chromatosome sample before preparation of the cryo-EM grids.

### Preparation of chromatosome

27. Dissolve ∼ 5 mg H1 and ProTα protein powder in 0.5 mL nuclease-free water by rocking for 30 min, then mix with 0.5 mL gel filtration buffer B (20 mM Tris-HCl, pH 7.4, 1 mM EDTA and 600 mM NaCl).
28. Remove any aggregates by centrifugation at 27,000x*g*, 4°C for 15 min. Inject the supernatant onto a Superdex S75 10/300GL Increase column that has been pre-equilibrated in gel filtration buffer B (20 mM Tris-HCl, pH 7.4, 1 mM EDTA and 600 mM NaCl). Run at 0.75 mL/min using an AKTA FPLC, collect the peak fractions and measure the concentration of each protein.
29. Cast a 4.8 % native PAGE gel (see materials and equipment). Pre-run the gel in 0.2x TBE at 120V, 4°C for 2 h.
30. Dilute nucleosome, ProTα, and H1 with TEN10 buffer (10 mM Tris-HCl, pH 7.4, 1 mM EDTA, 10 mM NaCl and 0.5 mM TCEP) to a concentration of 1, 2, and 1 uM, respectively. Titrate 100 nM nucleosome with 50, 75, 100, 125, 150, 175, or 200 nM of H1 in the presence of 200 nM ProTα and 2% Ficoll 400. Adjust the final volume of the binding reaction to 10 μL and incubate at 25°C for 15 min.
31. Load 5 μL of each reaction on a 4.8% native PAGE gel, run in 0.2x TBE buffer at 100 V for 120 min at 4°C.
32. Stain the gel with Midori Green Advance stain, visualize the gel using a ChemiDoc gel imaging system (Figure 5).

**Figure 5:**
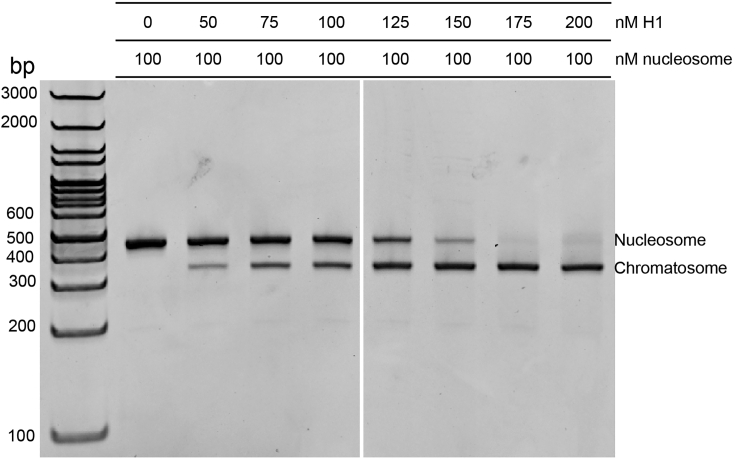
Titration of 100 nM nucleosome with various concentration of H1 resolved in a 4.8% native PAGE gel
33. Determine the best ratio for the nucleosome and H1. Use that ratio to make a large volume (>100 μL) of chromatosome by titration of the nucleosome with H1, in the presence of ProTα. Troubleshooting 4

**CRITICAL:** Mixing H1 and nucleosome at a high concentration (>100 uM) can cause aggregation. To prevent sample loss, dilute both the nucleosome and H1 to a concentration below 10 uM before titration or mixing.

### Preparation of scFv stabilized chromatosomes

34. Dilute scFv with TEN10 buffer to a concentration of around 0.5 uM, add 3x molar ratio of scFv to the chromatosome sample and incubate on ice for 15 min.
35. To remove extra scFv and ProTα, concentrate the complex to around 500 μL and inject onto a Superose 6 10/300GL column pre-equilibrated with TEN10 buffer. Run at 0.5 mL/min using an AKTA FPLC. Collect the peak fraction and concentrate to ∼100 μL using an Amicon Ultra 30K MWCO centrifugal filter unit.

### Vitrification of scFv stabilized chromatosome and screen grids using cryo-TEM

36. Measure the concentration of the scFv-bound chromatosome. Adjust the concentration to 4–5 uM with TEN10 buffer for sample vitrification.
37. Prepare liquid nitrogen cooled ethane and set the operation parameters of the Vitrobot Mark IV (Thermo Fisher Scientific) to 4°C, 100% humidity, 3 s blotting time.
38. Pretreat Quantifoil 1.2/1.3 holy carbon copper grids at 15 mA for 60 s using an easiGlow Glow Discharge Cleaning System.
39. Apply 3 μL of the chromatosome sample to the grid, blot and vitrify the grid using the Vitrobot. Transfer and store the grid in a liquid nitrogen tank.
40. For the latest generation of electron microscope with an autoloading system, assemble the grid with a clip ring and a C-shape spring in the “auto-grid” cartridge system.
41. Insert the grid into the electron microscope by the autoloader, screen the grids using SerialEM (Mastronarde, 2005). Troubleshooting 5

***Optional:*** For a traditional electron microscope with side-entry, chill the workstation with liquid nitrogen, carefully transfer the grid to the tip the cryo-holder and secure the grid with the clip ring. With the grid covered by the shutter, promptly insert the holder into the microscope from the side-entry. The grid is then ready for screening.

## Expected outcomes

**Figure 6:**
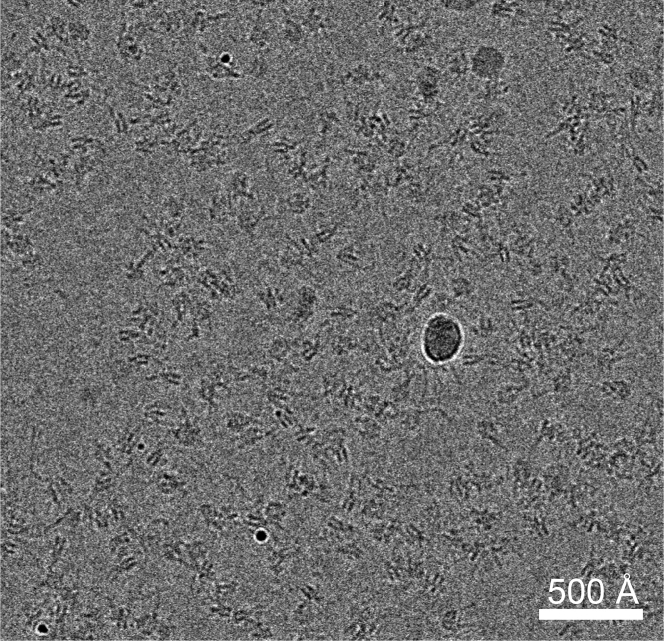
Typical cryo-EM micrograph of the scFv-chromatosome complex Scale bar, 500 Å.

Sample dissociation during the blotting and vitrification process hinders the study of many protein complexes by cryo-EM. In this protocol, we use scFv to stabilize the chromatosome (a nucleosome bound to a histone H1), which prevents chromatosomes from being absorbed to the water-air interface. Thus the whole complex remains intact in the vitrified ice. Our protocol does not rely on chemical crosslinking that many groups use to stabilize the nucleosome or its complexes. It can be broadly used to study the nucleosome with a natural DNA sequence or any nucleosome protein complex, provided that the scFv does not interfere with protein nucleosome interaction. Typical cryo-EM micrograph of the scFv stabilized chromatosome complex is shown in Figure 6.

## Limitations

Our protocol has the following limitations: scFv binds to the nucleosome acidic patch, which limits its application to nucleosome complexes in which proteins also interact with the acidic patch of the nucleosome. The procedure for purification of scFv may not apply to other scFv, as the charge property of each scFv may be different. For example, if the scFv has a very low isoelectric point (PI), then one should consider using anion resins instead of cation resins for ion exchange purification.

## Troubleshooting

### Problem 1

scFv does not bind to the SP beads.

### Potential solution

Carefully check the pH of the scFv refolding solution, make sure the pH is around or slightly below 7.8.

### Problem 2

Purified scFv has two bands on the SDS PAGE gel.

### Potential solution

Folded scFv with correct disulfide bonds runs slightly slower than misfolded scFv on the SDS PAGE gel (Figure 4). Try to rerun the Superdex S75 column and collect smaller fractions to better separate the folded scFv from the misfolded scFv.

### Problem 3

scFv yield is too low.

### Potential solution

Multiple factors could affect the yield of scFv:

At the denaturation step, make sure that the cysteines in the scFv are in reduced form by adding 1,4-DTE powder fresh to the denaturation buffer. DTE should be stored at −20°C as it is prone to oxidation at higher temperatures.

At the refolding step, add oxidized glutathione right before adding the denatured scFv solution. Limit the refolding time to at least 36 h, as refolding time that is too short or too long (> 1 week) will result in a low yield of the correctly refolded scFv.

At the purification step, note that scFv is not stable at 25°C, especially when it is not pure. Try to do all the purification steps at 4°C and chill all the buffers before use.

At the concentrating and storage steps, do not over-concentrate scFv, as it is not stable at concentrations that are higher than 10 uM. It is best to store it at a concentration of ∼ 5 uM in physiological buffer conditions and at 4°C. Freeze and thaw are not recommended.

### Problem 4

Loss of chromatosome during large scale preparation.

### Potential solution

The ratio of H1 to nucleosome should be carefully determined from the 4.8% native PAGE gel (Figure 5). When the number of H1 molecules is more than the nucleosomes, it binds to nucleosomes nonspecifically and causes aggregations. Dilute both the H1 and nucleosome to a concentration of 10 uM or less using TEN10 buffer. Generally, a step-by-step titration of nucleosome with H1 will help.

### Problem 5

Cryo-EM screen shows complex dissociation, even for scFv stabilized nucleosome or chromatosomes

### Potential solution

This happens when the vitrified ice is super thin. Try different blotting times and use a different kind of grid such as a Lacey grid.

## Resource availability

### Lead contact

Further information and requests for resources and reagents should be directed to and will be fulfilled by the lead contact, Yawen Bai (baiyaw@mail.nih.gov).

### Materials availability

Unique and stable reagents generated in this study are available upon request.

### Data and code availability

This protocol did not generate datasets or code.
